# Divergent brain functional network alterations in dementia with Lewy bodies and Alzheimer's disease

**DOI:** 10.1016/j.neurobiolaging.2015.05.015

**Published:** 2015-09

**Authors:** Luis R. Peraza, John-Paul Taylor, Marcus Kaiser

**Affiliations:** a Institute of Neuroscience, Campus for Ageing and Vitality, Newcastle University, Newcastle upon Tyne, UK; b Interdisciplinary Computing and Complex BioSystems (ICOS) research group, School of Computing Science, Newcastle University, Newcastle upon Tyne, UK

**Keywords:** Connectome, Brain networks, Cognitive fluctuations, Attention impairment, Resting-state

## Abstract

The clinical phenotype of dementia with Lewy bodies (DLB) is different from Alzheimer's disease (AD), suggesting a divergence between these diseases in terms of brain network organization. To fully understand this, we studied functional networks from resting-state functional magnetic resonance imaging in cognitively matched DLB and AD patients. The DLB group demonstrated a generalized lower synchronization compared with the AD and healthy controls, and this was more severe for edges connecting distant brain regions. Global network measures were significantly different between DLB and AD. For instance, AD showed lower small-worldness than healthy controls, while DLB showed higher small-worldness (AD < controls < DLB), and this was also the case for global efficiency (DLB > controls > AD) and clustering coefficient (DLB < controls < AD). Differences were also found for nodal measures at brain regions associated with each disease. Finally, we found significant associations between network performance measures and global cognitive impairment and severity of cognitive fluctuations in DLB. These results show network divergences between DLB and AD which appear to reflect their neuropathological differences.

## 1 Introduction

Dementia with Lewy bodies (DLB) is an increasingly recognized cause of dementia, accounting for 4%–8% of dementia cases (McKeith et al., 2007). Diagnostically, it is characterized by 3 core symptoms: cognitive fluctuations, visual hallucinations, and parkinsonism (Calderon et al., 2001, Collerton et al., 2003). Alzheimer's disease (AD) accounts for 50%–70% of the cases (McKeith et al., 2007), with memory loss as the main presenting symptom (Calderon et al., 2001). Although substantial previous research has achieved a criteria for the clinical differentiation of DLB to other dementias such as AD (McKeith et al., 1996, McKeith et al., 2005), at early stages the clinical phenotypes of these 2 diseases can overlap (Tiraboschi et al., 2006, Troster, 2008). This encourages research on the understanding of the etiological mechanisms of both diseases.

A neuroimaging alternative that can be used to understand DLB is resting-state functional magnetic resonance imaging (rs-fMRI). During resting-state intrinsic brain connectivity manifests in a series of systems or networks such as the default mode network (Raichle et al., 2001). These resting-state networks are considered a functional reflection of structural brain connectivity (Beckmann et al., 2005), which can be altered by neurodegenerative diseases as evidenced in previous rs-fMRI studies (Binnewijzend et al., 2012, Greicius et al., 2004, Seeley et al., 2009).

One approach to studying brain's functional connectivity is by building a graph whose topological characteristics reflect neural communication among brain regions (Eguiluz et al., 2005, Wang et al., 2010). Such systems can be analyzed using graph theory, which provides a powerful series of tools that help to understand and describe the brain's complexity globally and regionally (Kaiser, 2011, Rubinov and Sporns, 2010, Sporns et al., 2004). Brain network analysis has been shown to be a reliable method across neuroimaging modalities (Buchanan et al., 2014, Wang et al., 2011), and substantial research has been accomplished for the study of AD (He et al., 2009, Stam et al., 2007, Tijms et al., 2013). However, to date there have been no comparable studies in DLB.

In the present study, we therefore sought to characterize how rs-fMRI brain networks are altered in DLB using graph theory and to contrast our findings against a healthy control group, as well as an AD cohort, to determine DLB-specific network alterations. Our hypotheses were that: (1) DLB patients would show altered network connectivity such as decreased small-worldness when compared with controls, as is commonly reported in the literature for AD; (2) DLB and AD patients would have different topographic network alterations given their differing etiological bases, and thus, we would expect different patterns of expression in local network measures between the diseases; and (3) cognitive and core symptoms in DLB would map onto alterations in network measures.

## 2 Methods

### 2.1 Participants and assessment

The study involved 63 participants; 22 diagnosed as DLB, 24 as AD, and 17 healthy controls. Patients were recruited from the local population who had been referred to local old age psychiatry and neurology services. Diagnostic classification of patient groups was carried out by 2 independent experienced clinicians. Participants with AD fulfilled the National Institute of Neurological and Communicative Diseases and Stroke/Alzheimer's Disease and Related Disorders Association criteria for probable AD (McKhann et al., 2011). DLB patients met criteria for probable DLB, including the presence of 2 of 3 core symptoms; cognitive fluctuations, visual hallucinations, and parkinsonism (McKeith et al., 2005). Eleven out of the 22 DLB patients had dopaminergic imaging, and of these all had reduced bilateral uptake of tracer within their striata. Clinical assessment included the Mini-Mental State Examination (MMSE), Cambridge Cognitive Examination (CAMCOG), Unified Parkinson's disease rating scale (UPDRS) (Fahn and Elton, 1987). Caregivers were asked to complete the Clinical Assessment of Fluctuations (CAF) (Walker et al., 2000), and the Neuropsychiatric Inventory (NPI) (Cummings et al., 1994), with a particular focus on the hallucinations subscale (NPI^hall^) from a visual hallucinations perspective. Most of the patients were taking acetylcholinesterase inhibitors (AChEI; 21 DLBs and 24 Alzheimer's). The control group had no history of psychiatric or neurological brain disease and a MMSE score >27. Approval for this study was granted by the Newcastle Ethics Committee and all participants gave informed consent.

### 2.2 Imaging preprocessing and time series extraction

For the resting-state, participants laid within the magnetic resonance imaging scanner with eyes open and a total of 128 functional magnetic resonance imaging (fMRI) volumes were acquired (repetition time = 3000 ms). fMRI preprocessing was implemented using the FMRIB's Software Library (FSL 4.1; www.fmrib.ox.ac.uk/fsl). It included FMRIB's Linear Image Registration Tool for motion correction with spatial smoothing full width at half maximum of 6.0 mm, and high pass filter of 150 seconds. Structural and functional MRIs were nonlinearly coregistered to standard space Montreal Neurological Institute using FMRIB's nonlinear image registration tool. Motion parameters were analyzed for movement exclusion criteria (translations >2 mm and rotations >1°) as in Liao et al., 2010, Ni et al., 2012, and comparisons between groups for motion/rotation were evaluated by the motion/rotation formula (Liao et al., 2010):

$$\text{head}\;\text{motion/rotation}={\left(M-1\right)}^{-1}\sum_{i=2}^{M}\sqrt{{\left|x_{i}-x_{i-1}\right|}^{2}+{\left|y_{i}-y_{i-1}\right|}^{2}+{\left|z_{i}-z_{i-1}\right|}^{2}\text{,}}$$

where *x, y,* and *z* are the 3 motion/rotation parameters and *M* is the fMRI length.

To further clean the fMRI data sets, Multivariate Exploratory Linear Optimized Decomposition into Independent Components was carried out on each of the participant's fMRIs using standardized cleaning procedures (Kelly et al., 2010). Components resembling artefacts such as movement and cerebrospinal fluid, or whose power spectra were widespread through all frequencies or above 0.10 Hz were filtered out. Time series extraction was then implemented using 6-mm spheres from 100 cortical and 4-mm spheres from 12 subcortical regions of interest (ROIs). ROI coordinates were obtained from the Harvard-Oxford cortical/subcortical atlas in FMRIB's Software Library in standard MNI space. Then, the MNI coordinates were spatially transformed to fMRI participant space using the coregistration parameters. With the fMRI time series, Pearson correlation matrices were computed.

**Fig. 1:**
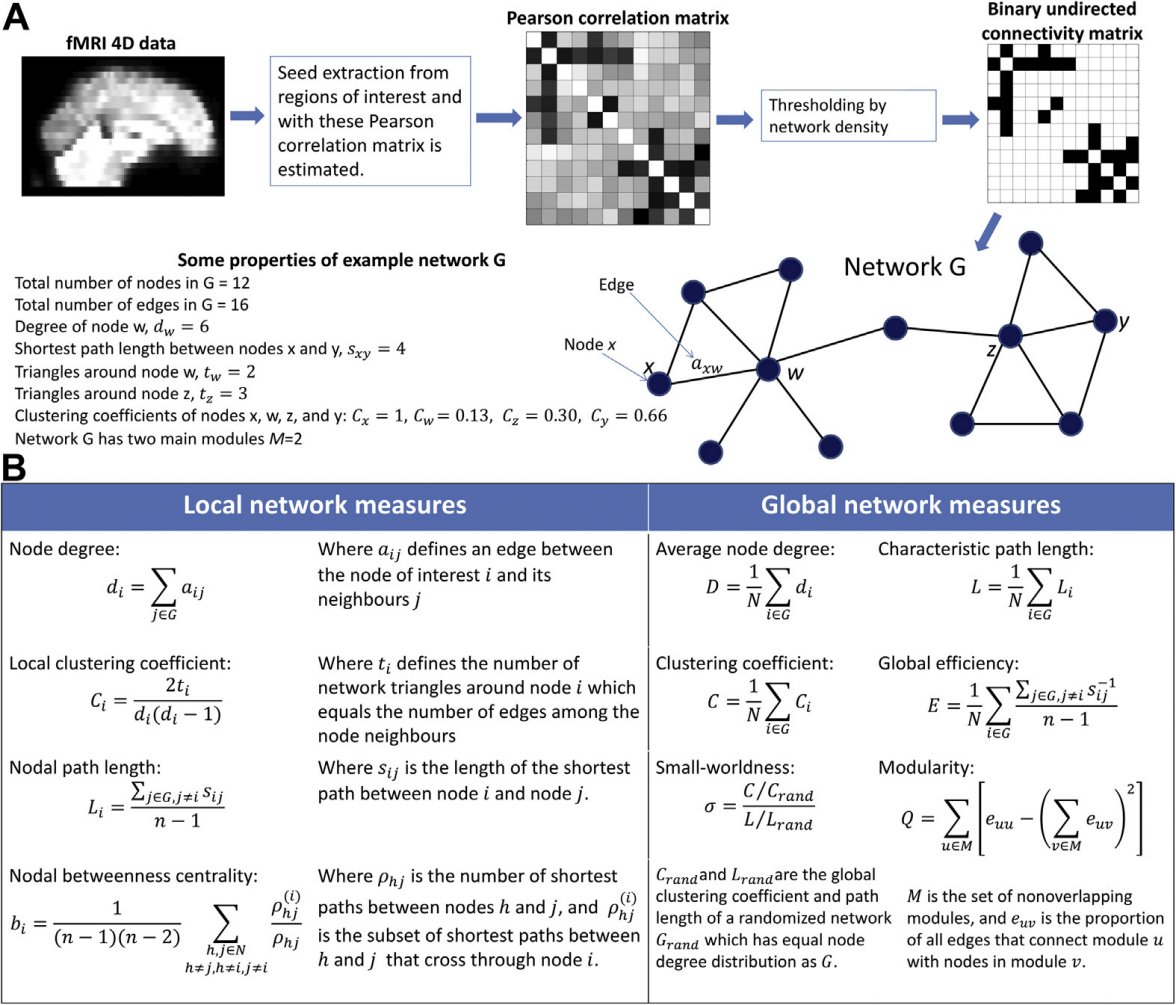
Functional network inference and network scores for unweighted, undirected connectivity matrices. (A) A weighted connectivity matrix is inferred from fMRI time series, then thresholded to a desired edge density or average node degree where the surviving edges become 1 second and the rest 0 seconds. Then, network scores can be applied to the resultant network. (B) Network measures used to characterize functional brain networks in dementia with Lewy bodies and Alzheimer's disease patients shown in local and global versions. All network measures were estimated using the Brain Connectivity Toolbox (Rubinov and Sporns, 2010). Abbreviation: fMRI, functional magnetic resonance imaging.

Previous investigations in rs-fMRI and Lewy body diseases have found the presence of functional anticorrelations in DLB (Galvin et al., 2011) and Parkinson's disease (PD) (Hacker et al., 2012). Because of this, and because we were interested in assessing brain connectivity regardless of whether it was positive or negative in our patient groups (De Vico Fallani et al., 2014), we took the absolute value of the correlation matrix for further graph analysis (Li et al., 2013). A schematic diagram showing the network inference steps is shown in Fig. 1A. ROIs, their coordinates and labels are listed in Supplementary Table 1.

### 2.3 Graph analysis

Brain networks are represented by graphs where ROIs are nodes and their interactions are edges or connections. We opted for the analysis of binary connectivity matrices obtained from the correlation matrices by thresholding these by edge density (van Wijk et al., 2010). This avoids obvious differences in the strength of the connectivity values between patients and controls, and focuses on the comparison of functional brain networks (De Vico Fallani et al., 2014).

Different measures have been developed to characterize network topologies. Network measures used in our study are briefly explained in Fig. 1B and further explained in previous published tutorials (Kaiser, 2011, Newman, 2003, Rubinov and Sporns, 2010, Stam and Reijneveld, 2007). All network measures in our study were estimated using the Brain Connectivity Toolbox (Rubinov and Sporns, 2010) in Matlab (MATLAB 7.14, The MathWorks Inc, Natick, MA, USA). With this, a battery of 3 analyses was implemented to study functional networks in our participants as explained in the following paragraphs:

#### 2.3.1 Edge strength and distance analysis

To compare topological differences between groups, network edges in all participants were classified as short, middle, and long, depending on the spatial Euclidean distance between 2 directly connected nodes. The boundaries for the distance range classification were chosen by identifying the longest and shortest possible distance between 2 nodes and dividing their difference into 3 equal ranges (short 2.82–56.63 mm, middle 56.63–110.43 mm, and long 110.43–164.23 mm according to the MNI space). For group comparisons, networks were thresholded at a range of network densities from 3.6% to 39.3%, in agreement to previous brain connectivity studies (Bohr et al., 2012, Gießing et al., 2013, van Wijk et al., 2010). Subsequently, edge strength and number of edges at each distance range were compared between groups.

#### 2.3.2 Local network measures

Three nodal network measures were studied: node degree, nodal clustering coefficient (Watts and Strogatz, 1998), and nodal betweenness centrality (Freeman, 1978). Node degree provides a measure of the number of edges connected to each node, where 2 nodes are said to be neighbors when directly connected by an edge, and nodal betweenness centrality measures the number of shortest paths that cross a node. A region with high node degree is highly connected to other regions, whereas a region with high betweenness centrality would be a region easily reached by distant parts of the network. Nodal clustering coefficient measures the ratio of a node's connected neighbors relative to the maximum number of connections among them. Brain functional networks often show high clustering coefficient because neuronal communication among cortical regions tends to arrange in communities (Fair et al., 2009, Newman, 2006b).

#### 2.3.3 Global network measures

Five global network measures were studied: characteristic path length *L* (Watts and Strogatz, 1998), clustering coefficient *C*, global efficiency *E* (Latora and Marchiori, 2001), modularity *Q* (Newman, 2006a, Newman, 2006b), and small-worldness *σ* (Humphries and Gurney, 2008). *L* measures the average shortest path between any 2 nodes within the network. *C* is the average of all nodal clustering coefficients. *E* is the average inverse of *L*, and it is indicative of efficient communication among brain nodes or communities. *Q* measures how separated brain communities are among themselves; previous research has shown that the functional brain tends to be modular (Meunier et al., 2010). Small-worldness measures how well-connected the brain network is both locally and globally (Power et al., 2010), and it is estimated as the ratio between the normalized clustering coefficient *C*_*norm*_ and the normalized characteristic path length *L*_*norm*_. This normalization is achieved by dividing *C* by the clustering coefficient of a random network *C*_*rand*_ of equal node degree distribution leading to *C/C*_*rand*_, and similarly for *L*_*norm*_; *L/L*_*rand*_ (Humphries and Gurney, 2008). To estimate *C*_*rand*_ and *L*_*rand*_, we used the average indices of 50 random networks preserving the node distribution of the real networks (Sanz-Arigita et al., 2010). A small-worldness higher than 1 means that the network showed efficient connectivity (Sporns et al., 2004). Previous research consistently reports that the brain has a small-world network architecture (Sporns et al., 2004, Stam and Reijneveld, 2007, van den Heuvel et al., 2008) and that the brain's small-worldness is altered in AD, probably due to network disconnections driven by neural degeneration (Delbeuck et al., 2003). We also report *C*_*norm*_ and *L*_*norm*_ as part of our results.

### 2.4 Statistical analysis

Statistics for demographic and clinical variables were performed using Statistical Package for Social Sciences (version 22). Head motion and rotation were assessed in Statistical Package for Social Sciences by 2 Kruskal-Wallis tests. Connectivity values between groups for short, middle, and long edges were assessed by 3-level analyses of variance (*p*-value < 0.05) with post hoc Bonferroni corrected (*p*-value < 0.05/3) unpaired *t* tests in Matlab. Significant differences for global network measures were assessed using 3-level analysis of variance (*p*-value < 0.05) with post hoc Bonferroni corrected unpaired *t* tests (*p*-value < 0.05/3, Matlab) along all network densities. We also estimated integrated global network measures by averaging their values through all assessed edge densities (Ginestet et al., 2011), and tested for statistical differences using 2-tailed unpaired *t* tests. This integration solves the multiple comparisons problem caused by assessing several edge densities (Gießing et al., 2013). Comparisons for local network measures were assessed with 2-tailed unpaired *t* tests (*p*-value < 0.05 uncorrected). The number of times significance was reached per node and edge density was measured by its nodal consistency *S*_*i*_ (Zhao et al., 2012), which provides an exploratory measure of local network differences between groups (see Supplementary Material). Finally, integrated network measures were compared with clinical scores (MMSE, CAMCOG, CAF, NPI^hall^, and UPDRS) using Spearman's rank correlations (Matlab).

## 3 Results

Of the 63 participants, 2 AD patients were excluded from the study because of coregistration problems. Additionally, 4 DLB and 3 AD patients were also excluded due to excessive head motion and/or rotation (>2 mm translation). The motion and/or rotation parameters of the remaining cohort (18 DLBs, 19 ADs, and 17 healthy controls) were <2 mm for translation and <1° for rotation. Furthermore, head motion and/or rotation differences were not significant between groups (Supplementary Material).

### 3.1 Demographic and clinical measures

Statistical comparisons of demographic and clinical variables are shown in Table 1. The 3 groups were matched for age, with DLB and AD patients matched for MMSE and CAMCOG scores. Both patient groups were more cognitively impaired than controls. Cognitive fluctuation severity and frequency assessed by the CAF were higher in DLB than AD. For the NPI, both patient groups were matched in terms of the overall score, but DLB patients had higher subscale scores (NPI^hall^) for the frequency and severity of hallucinations.

### 3.2 Spatial distance and strength of connections

**Fig. 2:**
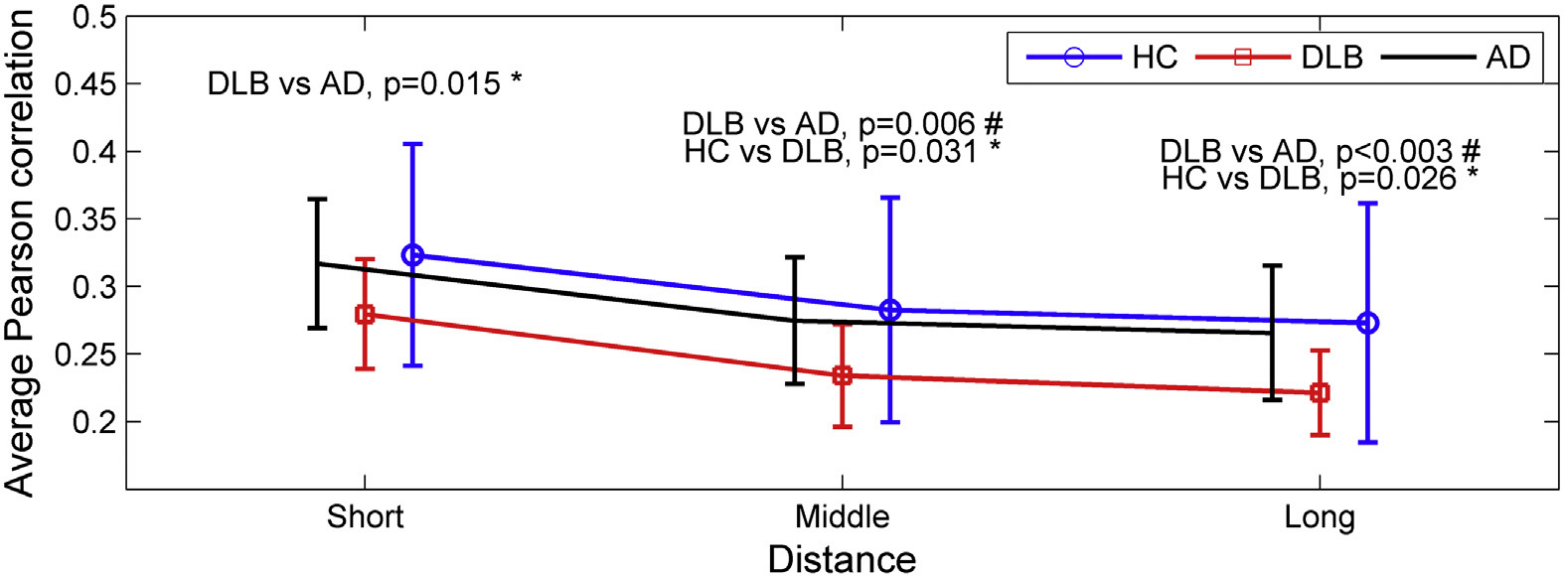
Connectivity strength analysis between groups. Mean correlation strength for the 3 groups (healthy controls [HC], dementia with Lewy bodies [DLB], and Alzheimer's disease [AD]) at 3 edge distance ranges (short, middle, and long) according to their Euclidean distance between nodes. Error bars indicate 1 standard deviation from the mean. As expected, the control group showed higher mean connectivity strength, while the 2 patient groups showed lower correlation strength. “#” stands for results that were significant after analysis of variance test (*p*-value < 0.05) with post-hoc Bonferroni correction (*p*-value < 0.05/3). “*” stands for significant results at *p*-value < 0.05, 2-tailed unpaired *t* test, uncorrected.

Fig. 2 shows the average correlation for 3 edge distance ranges (short, middle, and long). Correlation strength was lower in DLB compared with AD for all 3 ranges (*p*-value < 0.015). Similarly, correlation strength in DLB was significantly lower than controls for middle and long edges (*p*-value < 0.05); for AD versus controls, edge strength was not significantly different for any of the 3 ranges.

Additionally, the number of edges was counted at each network density for each participant. The number of middle and long-range edges in the 3 groups was not significantly different. However, when studying the presence of short edges, the number of these were significantly higher in the DLB group compared with AD (*p*-value < 0.05) at network densities 17.1%, 18.9%, and 21.6% (Supplementary Material).

### 3.3 Global network measures

**Fig. 3:**
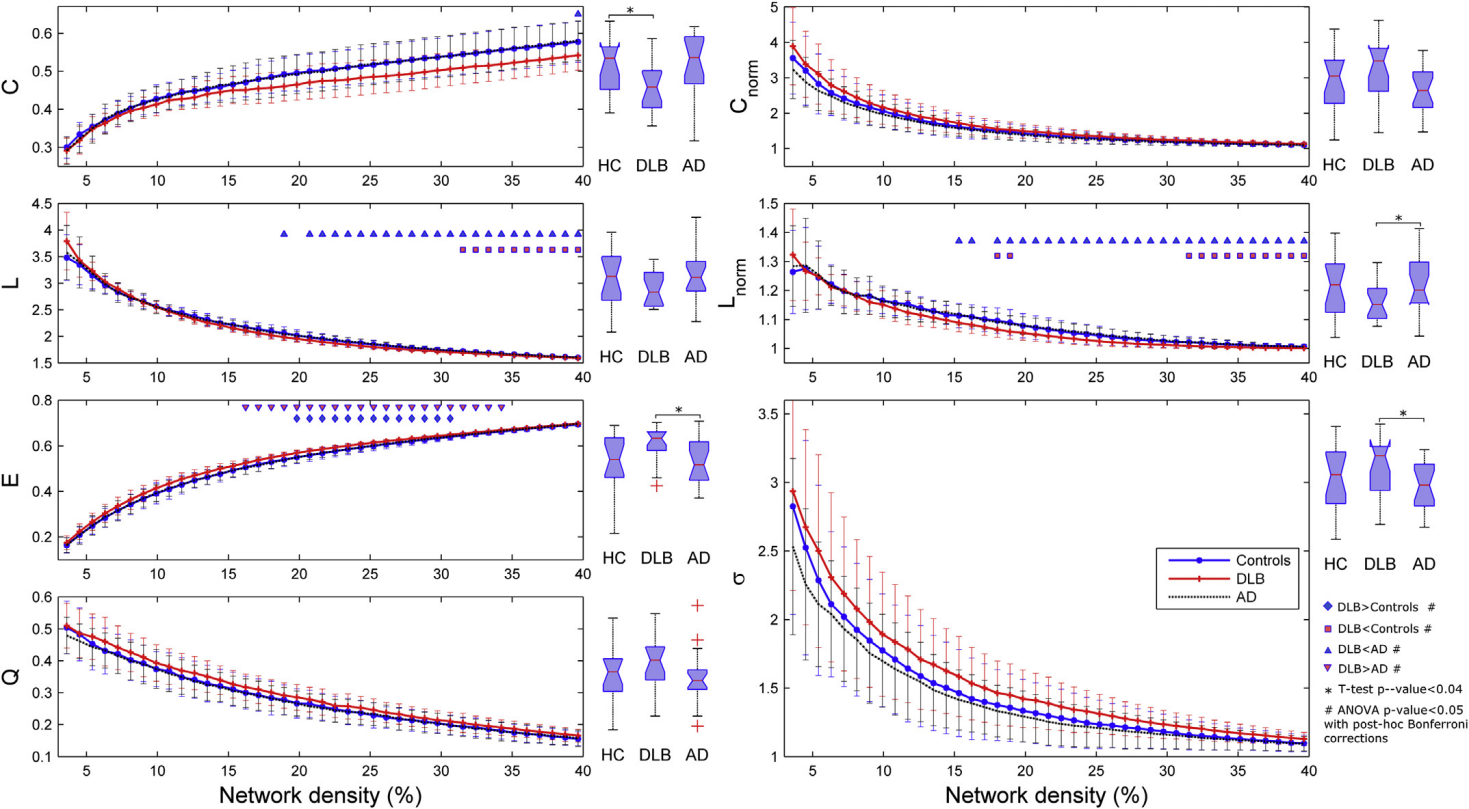
Comparisons between groups for global network measures at different edge densities from 3.6% to 39.6% (shown as curves) and using integrated network measures, that is, average across this range of edge densities (shown as box plots at the right of each network measure's curve). Measures assessed were: clustering coefficient *C*, characteristic path length *L*, global efficiency *E*, modularity *Q*, normalized clustering *C*_*norm*_, normalized path length *L*_*norm*_, and small-worldness *σ*. On average, dementia with Lewy body (DLB) patients showed higher *E* and lower *C* compared with healthy controls (HC) and Alzheimer's disease (AD) patients. Small-worldness is also higher in DLB compared with AD. Error bars indicate 1 standard deviation from the mean. Triangular and squared markers are indicative of significant differences (analysis of variance [ANOVA] with post-hoc Bonferroni correction) between studied groups at the indicated edge densities. Asterisks (*) show significant differences (2-tailed unpaired *t* tests) between the indicated groups using integrated network measures estimated from each participant.

Statistical comparisons for the global network measures (integrated and nonintegrated) are shown in Fig. 3. The DLB group showed higher global efficiency *E* than the AD and healthy controls. Significant differences for *E* were obtained between DLB and AD patients at densities 16.2%–34.2% and for DLB versus controls at 19.8%–30.6%. AD and controls did not show significant differences for *E*. The characteristic path length *L* was significantly lower in DLB compared with controls and Alzheimer's at densities 31.5%–39.6% and 18.9%–39.6%, respectively. Modularity *Q* did not show significant differences, but there was a trend for higher modularity in DLB and lower modularity in AD compared with controls.

The normalized clustering coefficient *C*_*norm*_ showed different results to the non-normalized score: *C*_*norm*_ was on average higher in DLB than in controls and Alzheimer's patients for the entire range edge densities. The normalized characteristic path length *L*_*norm*_ was significantly higher in AD than in DLB patients for densities 15.3%–39.6%. Small-worldness *σ* did not show significant differences, but it was on average higher in DLB than in controls and AD.

When integrated across the range of edge densities, global network measures still showed significant differences (box plots in Fig. 3). The DLB group showed lower *C* than healthy controls (*p*-value = 0.036) and lower *L*_*norm*_ compared with AD (*p*-value = 0.024). Also, DLB showed higher integrated *E* (*p*-value = 0.018) and *σ* (*p*-value = 0.038) than AD patients.

### 3.4 Local network measure comparisons

**Fig. 4:**
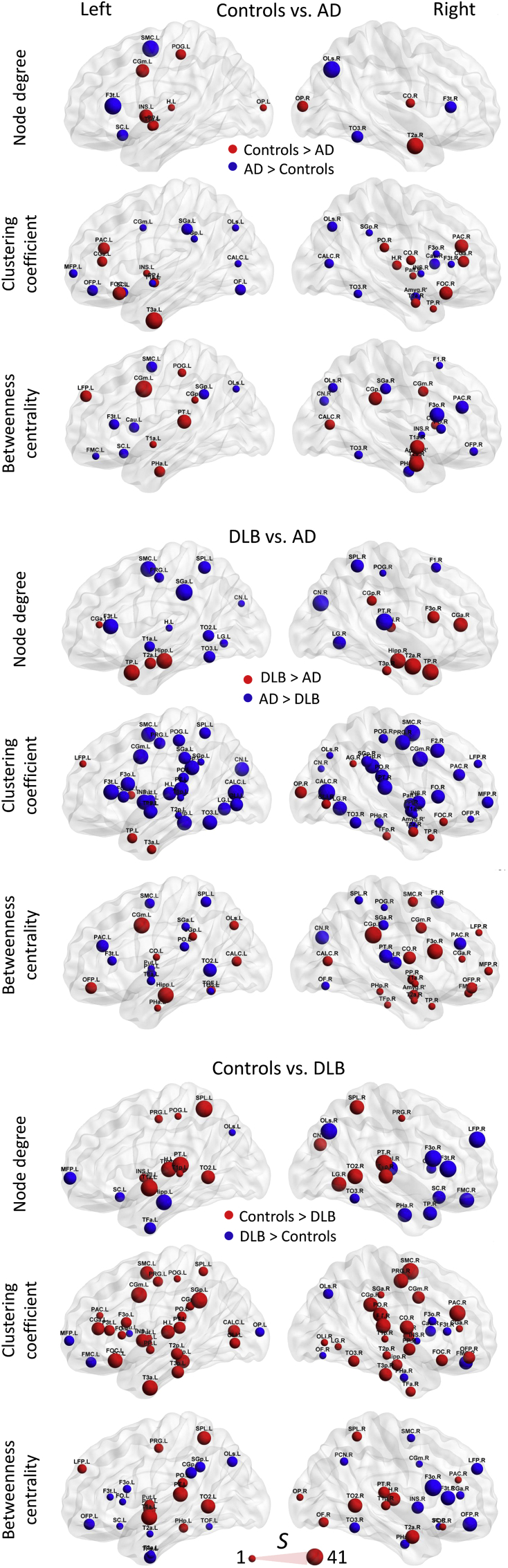
Local network measure comparisons; node degree, clustering coefficient, and betweenness centrality for comparisons between groups; controls versus Alzheimer's disease (AD), Controls versus dementia with Lewy bodies (DLB), and DLB versus AD. Spheres are proportional to the consistency value *S* through all edge densities with a minimum value of *S* = 1 and maximum of *S* = 41 if the difference was significant at all densities from 3.6% to 39.6%. For node names and a list of consistency values, see Supplementary Tables 1–4. Brains were plotted using BrainNet Viewer (Xia et al., 2013).

Each brain plot in Fig. 4 shows network nodes with significantly different nodal measure values (*p*-value < 0.05) evaluated through all edge densities. The size of the spheres is proportional to the nodal consistency *S*_*i*_ (Zhao et al., 2012) for significant differences across densities, that is, bigger spheres indicate that the region was significant in terms of nodal consistency *S*_*i*_ at several densities with a maximum value of *S*_*i*_ = 41, if the difference was significant at all densities; 3.6%–39.6% (equivalent to an average node degree from 4 to 44 edges per node; see Supplementary Material).

Regional differences were found by the 3 local network measures for DLB versus AD. DLB patients showed higher node degree in temporal lobes compared with AD, including both hippocampi, while nodes with lower degree were found at posterior areas (parietal and occipital) in DLB patients. In addition, the right frontal lobe showed nodes with higher node degree in DLB when compared with AD. For nodal clustering coefficient, widespread brain areas covering frontal, parietal and occipital lobes showed lower scores in DLB. In contrast to DLB, AD patients demonstrated lower clustering coefficient in temporal cortices and the right occipital pole. Betweenness centrality showed less regionalized differences between the patient groups.

Comparisons for controls versus DLB patients on node degree revealed lower scores in DLB primarily at parietal and posterior temporal cortices. Nodal clustering coefficient was lower in DLB compared with controls in most of the brain cortex, with fewer differences in occipital lobes. In DLB both thalamic nodes showed higher node degree, and the left hippocampus showed lower node degree compared with controls. Betweenness centrality demonstrated lower scores at temporal and parietal regions in DLBs compared with controls.

Comparisons between controls and AD patients showed fewer differences than controls versus DLB. AD patients showed lower scores for node degree and clustering coefficient primarily at both temporal poles and frontal lobes. Lower betweenness centrality was found in AD in temporal regions, cingulate gyri, and superior parietal lobules.

### 3.5 Correlation with clinical measures

**Fig. 5:**
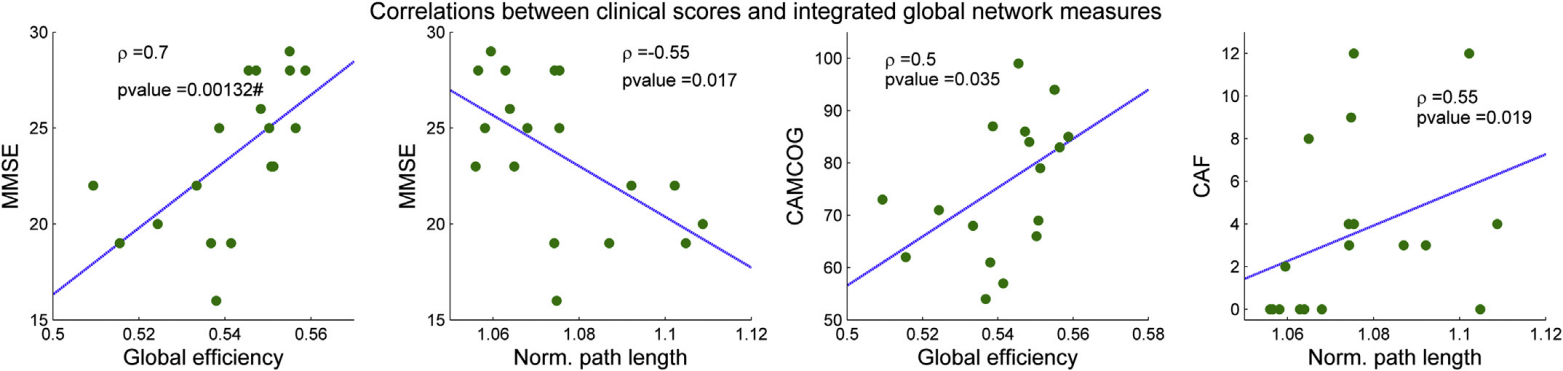
Spearman's rank correlations between clinical scores in dementia with Lewy body (DLB) and integrated global network measures (the network measure average estimated along all edge densities) in the same group. The MMSE and CAMCOG scores, both measures of cognitive impairment, showed a positive significant correlation with global efficiency. Furthermore, the MMSE showed a negative correlation with the normalized characteristic path length. The CAF score, which measures the level of cognitive fluctuations in patients with DLB, also showed a significant positive correlation with the normalized characteristic path length. “#” stands for results that survived Bonferroni correction (*p*-value < 0.05/35), while the rest of the significant correlations are shown as uncorrected (*p*-value < 0.05). Abbreviations: CAF, Clinical Assessment of Fluctuations; CAMCOG, Cambridge Cognitive Examination; MMSE, Mini-Mental State Examination.

For DLB, significant correlations were found between MMSE, CAMCOG, and CAF and integrated global efficiency *E* and normalized path length *L*_*norm*_ (Fig. 5), although the correlation between MMSE and *E* is the only result that survived Bonferroni correction. MMSE and CAMCOG are positively correlated with *E* and the MMSE is negatively correlated with *L*_*norm*_ in DLB. The CAF score, which ranges from 0 (no cognitive fluctuations) to 12 (maximum level of cognitive fluctuations in our group) showed a positive correlation with *L*_*norm*_. We could not find significant correlations between AD clinical scores and integrated network measures. We did, however, find a positive correlation trend in the AD group between clustering coefficient and MMSE-CAMCOG, and between small-worldness and CAMCOG at low edge densities (Supplementary Material).

## 4 Discussion

In this study, we found significant differences in the functional brain network of DLB patients compared with AD and healthy controls. First, network alterations in DLB were broader than in AD. Second, both patient groups showed divergent network alterations manifested in the integrated global measures. For instance, DLB patients showed higher small-worldness, while AD patients showed lower small-world index when both groups were compared against healthy controls. Finally, we found significant correlations between cognitive and fluctuating attention clinical scores and global network measures in DLB.

### 4.1 Network edge differences in DLB, AD, and healthy controls

Previous studies indicate that connectivity strength tends to be lower in AD (Binnewijzend et al., 2012, Greicius et al., 2004, Liu et al., 2013); the lack of significant difference in connectivity strength between Alzheimer's and healthy controls in the present study might be attributed to the mild stage of our Alzheimer's group with a relatively high MMSE score (mean, 22.58; standard deviation, 2.9) compared with other studies (Liu et al., 2013, Zhao et al., 2012). In support of this, Liu et al., 2013 found strength differences for long distance connections in a severely impaired AD group (MMSE mean, 6.2; standard deviation, 4.9) but not in less impaired participants. It is probable that during the early stages of neurodegeneration, compensatory responses are activated to maintain functional integrity (Backman et al., 2000). In DLB, the significantly lower functional synchronization might be associated with the presynaptic dysfunction caused by α-synuclein aggregates which are present in the brain cortex, even at early stages of the disease (Kramer and Schulz-Schaeffer, 2007, Schulz-Schaeffer, 2010). These presynaptic aggregates have been implicated in the impairment of neurotransmitter release which would manifest in lower synchronization of neuronal groups (Pogarell et al., 2006).

### 4.2 Altered small-world topology in AD and DLB

One important finding in this study, and contrary to our a priori hypothesis, was the observation of higher small-worldness in DLB compared with healthy controls. The AD cohort showed lower small-worldness than healthy controls, and this finding is in agreement with most of the previously published research (Sanz-Arigita et al., 2010, Stam et al., 2007, Supekar et al., 2008). A decrease of small-worldness has been suggested to arise as a result of a brain network randomization (Stam et al., 2009) consequent to the Alzheimer-related pathology.

The higher small-worldness we observed in DLB is driven by a higher normalized clustering coefficient which is indicative of higher local connectivity. Certainly, our results on edge distance comparisons suggest a relative increase of short edges in DLB compared with controls, which explains the higher clustering coefficient and the higher modularity *Q* shown in our results. The trend of higher *Q* in DLB indicates a dissociation of functional brain modules, suggesting that these are less connected. In contrast, lower *Q* in AD has previously been reported (Chen et al., 2013, Sun et al., 2014), and our results concur with this.

Increases in the number of short range edges in DLB are an effect of the threshold of the connectivity matrices by edge density (see Section 2). We observed higher desynchronization of distant brain regions in DLB as evidenced by significant low correlation strengths in middle and long distance connections (see Fig. 2). This results in networks with a more regular topology in DLB, with more connections to the nearest neighbors and more dissociated functional modules, which additionally leads to the effect of a higher normalized clustering coefficient and higher small-worldness. This phenomenon is explained in Telesford et al. (2011), where it is shown to be dependent on the edge rewiring of the real networks to estimate *C*_*rand*_ and *L*_*rand*_ (Telesford et al., 2011). We present detailed analyses on this in Supplementary Material.

Higher desynchronization of long distance connections have been reported using other neuroimaging modalities in Lewy body dementias (Bosboom et al., 2009, Kai et al., 2005), and this maps onto the known structural abnormalities in white matter in DLB (Kiuchi et al., 2011, Watson et al., 2012).

Neurobiological explanations for the network regularization in DLB remain speculative at this point but could include the significant known deficit in cholinergic function which occurs in DLB (Aarsland et al., 2004, Tiraboschi et al., 2000). Certainly, loss of cholinergic function may have a deleterious effect on long-range synchronicity (Bosboom et al., 2009, Kai et al., 2005); and secondly, deficits of this neurotransmitter might impair the ability of the cholinergic system to inhibit intracortical communication of close range neuronal groups, which is the mechanism by which the brain can enhance thalamocortical communication in the presence of external stimuli (Kimura et al., 1999, Lucas-Meunier et al., 2003, Picciotto et al., 2012, Proulx et al., 2014). From a network perspective, the impact of these changes could be the manifestation of a relative increase of cortical short distance connections in the functional network, producing a more regular network topology with higher small-worldness. Supporting evidence for this comes from a recent investigation from Baggio et al. (2014) in PD patients with mild cognitive impairment, whose functional brain network also presented higher small-worldness compared with healthy controls.

These ideas also resonate with our previous report in which we found functional disconnections between the frontoparietal attention system and basal brain regions associated with cognitive fluctuations in DLB (Peraza et al., 2014), a symptom which has been speculated to be dependent on cholinergic deficits (Ballard et al., 2001). However, we are cautious in over-interpreting our findings, as clearly a confounding element was the concurrent use at the time of scanning of AChEI in our patient groups; previous research has demonstrated functional connectivity can be restored in mild AD (Li et al., 2012) and PD patients (Possin et al., 2013). However, although to date there is no research work assessing influences in rs-fMRI by this medication in DLB, it is known that DLB patients have a much more severe deficit in cholinergic function compared with AD patients (Ballard et al., 2001, Lam et al., 2009). Thus, we would argue that it is likely that functional alterations arising as a result of cholinergic dysfunction are still likely to be evident in DLB despite AChEI treatment.

### 4.3 Regional network differences in DLB and AD

The lower node degree and nodal clustering in temporal cortices including both hippocampi in AD and parietal, occipital and frontal cortices in DLB as shown in Fig. 4, are consistent with the disease-specific regional predilections in pathology that have been demonstrated in previous neuroimaging and postmortem studies (Burton et al., 2004, Watson et al., 2012, Zhou et al., 2008). The higher node degree in both thalamic nodes in DLB compared with controls may reflect compensatory responses (Galvin et al., 2011, Kenny et al., 2012), and it is notable that thalamic alterations appear apposite to the significant attention deficits and cognitive fluctuations which occur in DLB (Delli Pizzi et al., 2014).

When comparing AD and control participants, regional differences are not as obvious as in DLB versus AD and DLB versus controls. Nevertheless, lower node degree, clustering coefficient, and betweenness centrality were evidenced, particularly in temporal cortices in AD, thus aligning with the tendency of the Alzheimer-related pathology to affect these structures early in the disease course (Firbank et al., 2010, Zhao et al., 2012).

### 4.4 Correlation with clinical scores in DLB

In the DLB group, the MMSE and CAMCOG scores, measures of global cognitive function, were correlated with the integrated global efficiency. In addition, the normalized path length and CAF score in DLB demonstrated a significant positive correlation, suggesting that inefficient connectivity may contribute to the manifestation of cognitive fluctuations in DLB. In contrast, we did not see any association between global measures and the other DLB core symptoms of parkinsonism or visual hallucinations. However, previous investigations suggest that complex visual hallucinations in DLB are triggered by attention and cognitive deficits (Collerton et al., 2005, Shine et al., 2011, Uchiyama et al., 2012), symptom domains whose clinical scores were correlated with global network metrics in the current investigation. A possible explanation might be that the symptoms of impaired cognition and attention are so inter-related with alterations in the functional brain network that these conceal significant relations with the symptoms of complex visual hallucinations and parkinsonism, and these are more regionally specific, for example, visual areas (Heitz et al., 2015, Taylor et al., 2012) and motor system (Huang et al., 2007). We conducted exploratory analyses which provided some tentative support for this conclusion (Supplementary Material).

We did not see any correlations with the clinical scores in the AD group. One explanation may be that our AD group was less cognitively impaired compared with previous reports, and because of this, we were only able to find correlation trends of MMSE and CAMCOG with network measures in AD. In DLB, cognitive impairment and fluctuations appear to depend on the degree of cholinergic dysfunction, and thus these findings provide further support for the inference that functional network alterations in DLB may depend on this neurotransmission system, although this must be taken with the caveat that, we had no direct measure of the degree of cholinergic dysfunction in our patients as well as the confound that most of our patients were taking AChEI.

## 5 Conclusions

Our results of decreased small-worldness and lower nodal network measures in temporal cortices in AD agree with the current consensus in the literature. In DLB, small-worldness was increased, which is in contrast to AD results and may be consequent to network regularization arising as a result of a relative loss of long-range connections. We further demonstrated that network alterations are associated with the degree of cognitive impairment and cognitive fluctuations in DLB, highlighting the potential of these metrics as disease biomarkers, as well as providing further insights into the pathoetiologic mechanisms in this condition. Future investigations in DLB and across the Lewy body disease spectrum should consider novel multimodality approaches; see for instance Whitwell et al. (2012) and Nombela et al. (2014), to better understand and characterize this dementia and its triggering mechanisms for an earlier and more accurate clinical diagnosis.

## Disclosure statement

The authors have no conflicts of interest to disclose.

## Figures and Tables

**Table 1:** Demographic, clinical, and cognitive measures

Demographic, clinical, and cognitive measures	DLB (n = 18)	AD (n = 19)	HC (n = 17)	*p*-value
Male:female	13:5	16:3	14:3	χ^2^ = 0.93, *p* = 0.62^a^
Age	77.2 ± 6.18	74.7 ± 8.5	76.8 ± 5.7	F_2,51_ = 0.671, *p* = 0.516^b^
MMSE	23.6 ± 3.9	22.58 ± 2.9	29.1 ± 0.85	t_35_ = 0.91, *p* = 0.36^c^
UPDRS	17.44 ± 7.8	1.74 ± 1.8	1.41 ± 1.87	t_34_ = 0.53, *p* = 0.59^d^
CAMCOG	76.2 ± 13.5	72.2 ± 11.5	96.4 ± 3.37	t_35_ = 0.97, *p* = 0.33^c^
CAF scale	3.29 ± 4.06^e^	0.61 ± 1.54^f^	na	t_33_ = 2.61, *p* = 0.006^c^
NPI total	8.71 ± 5.47^e^	6.33 ± 7.07^f^	na	t_33_ = 1.10, *p* = 0.277^c^
NPI hallucinations	1.65 ± 1.83^e^	0.0 ± 0.0^f^	na	t_33_ = 3.81, *p* < 0.001^c^

Values expressed as mean ± 1 standard deviation.

Key: AD, Alzheimer's disease; CAF, Clinical Assessment of Fluctuating Confusion; CAMCOG, Cambridge Cognitive Examination; DLB, dementia with Lewy bodies; HC, healthy controls; MMSE, Mini-Mental State Examination; na, not applicable; NPI, Neuropsychiatric Inventory; UPDRS, Unified Parkinson's Disease Rating Scale.

a χ^2^ test, DLB, AD, and HC.

b Analysis of variance DLB, AD, and HC.

c Student *t* test AD and DLB.

d Student *t* test HC and AD.

e (n = 17).

f (n = 18).
