# Supplementary material for: Divergent brain functional network alterations in dementia with Lewy bodies and Alzheimer's disease
Source: Neurobiol Aging. 2015 Sep;36(9):2458–67. doi: 10.1016/j.neurobiolaging.2015.05.015 (PMC4706129; doi:10.1016/j.neurobiolaging.2015.05.015)
Supplement: Supplementary Material [file mmc1.docx]

**Supplementary Material**

**1 MRI and fMRI acquisition**

**2 Motion and rotation parameters of the study cohort**

**3 Average node degrees and edge densities**

**4 Brain parcellation for connectivity analysis**

**5 Number of edges at the three edge ranges**

**6 Consistency S_i_ and results for S_i_>10**

**7 Correlation trends between cognitive scores in AD and network measures**

**8 Higher small-worldness in DLB compared to healthy controls and AD**

**9 Local correlations of Parkinsonism and visual hallucination clinical scores**

**1 MRI and fMRI acquisition**

Imaging was performed using a 3T Philips Intera Achieva scanner. Structural images were acquired with a magnetization prepared rapid gradient echo sequence (MPRAGE), sagittal acquisition, echo time 4.6ms, repetition time 8.3ms, inversion time 1250ms, flip angle = 8°, SENSE factor=2, in-plane field of view 240x240 mm with slice thickness 1.0mm, yielding a voxel size of 1.0x1.0x1.0mm. For resting state, participants laid within the scanner with eyes open and scans were obtained with a gradient echo-planar imaging (GE-EPI) sequence with 25 contiguous axial slices, 128 volumes, anterior-posterior acquisition, in-plane resolution = 2 × 2 mm, slice thickness = 6 mm, repetition time = 3000 ms, echo time = 40 ms, field of view = 260 × 260 mm. An axial orientation gradient echo T1 weighted image was also acquired to aid coregisteration of the resting state to the structural, TR 223 ms, TE 2.3 ms, flip angle 80°, slice thickness 4mm, pixel size 1.5x1.5 mm.

**2 Motion and rotation parameters of the study cohort**

Those patients that showed high movement, >2mm in translation and >1̊ in rotation, were excluded from the brain connectivity analysis. After these exclusion criteria, the remaining cohort of participants included 18 DLBs, 19 ADs and 17 healthy controls.

From these participants, the motion and rotation parameters estimated using the head motion/rotation formula ([Liao, et al., 2010](#_ENREF_10)) were: AD motion, mean 0.1390 with standard deviation (SD) 0.078, rotation mean 0.0015 SD 0.0014; DLB motion, mean 0.119 SD 0.0479, rotation mean 0.0011 SD 0.0005; Healthy controls, motion mean 0.122 SD 0.059, rotation mean 0.00114 SD 0.00083. Motion parameters are given in millimetres and rotation in radians and these parameters were not significantly different between the three groups (Kruskal-Wallis H test: motion p-value=0.98, χ^2^=0.031, df=2 and rotation p-value=0.387, χ^2^=1.9, df=2). The Kruskal-Wallis tests were performed in SPSS as explained in main document.

**3 Average node degrees and edge densities**

In our study we analysed brain connectivity in a range of edge densities from 3.6% to 39.7% in agreement with previous research which has reported that this range is of biological importance ([Bohr, et al., 2012](#_ENREF_1),[Gießing, et al., 2013](#_ENREF_8),[van Wijk, et al., 2010](#_ENREF_22)). Edge density (also described as wiring cost in the literature) is the proportion of edges in the network relative to the maximum possible number of edges. The range of densities chosen in our study is equivalent to an average node degree from 4 to 44 edges per node for a network of 112 nodes. We thresholded the connectivity matrices by average node degree with steps of one degree, giving a total of 41 steps. Results in the main document are reported in edge densities (%) instead of average node degrees as this is the convention in brain connectivity studies ([De Vico Fallani, et al., 2014](#_ENREF_6),[Power, et al., 2010](#_ENREF_12),[van Wijk, et al., 2010](#_ENREF_22)) and for reproducibility and comparisons of our results to previous studies.

**4 Brain parcellation for connectivity analysis.**

**Supplementary Table S1**. Regions of interest. Each hemisphere was parcelled in 56 regions following the cortical and subcortical Harvard-Oxford atlases in FSL.

| ROI (56) | Name of the region in the brain cortex | MNI coordinates | |
| --- | --- | --- | --- |
|  |  | Left hemisphere | Right hemisphere |
| AG | Angular gyrus | -50.45, -55.74, 29.3 | 52.15, -51.69, 32.16 |
| CALC | Intracalcarine cortex | -10.47, -74.92, 8.19 | 11.93, -73.63, 8.36 |
| CGa | Anterior Cingulate gyrus | -4, 4, 38 | 4, 4, 36 |
| CGm | Middle Cingulate gyrus | -4, 38, 10 | 4, 38, 10 |
| CGp | Posterior Cingulate gyrus | -6.3, -38.56, 28.79 | 6.96, -35.8, 30.04 |
| CN | Cuneal cortex | -8.66, -80.04, 27.62 | 9.38, -78.23, 27.98 |
| CO | Central opercular cortex | -48.03, -8.28, 11.64 | 49.47, -5.66, 11.1 |
| F1 | Superior frontal gyrus | -14.56, 17.96, 56.57 | 15.09, 17.55, 57.52 |
| F2 | Middle frontal gyrus | -38.15, 18.31, 42.01 | 39.19, 18.3, 43.02 |
| F3o | Inferior frontal gyrus | -50.69, 14.63, 15.22 | 52.49, 15.48, 16.37 |
| F3t | Inferior frontal gyrus | -49.76, 28.6, 8.59 | 51.84, 27.82, 7.72 |
| FMC | Frontal medial cortex | -5.38, 43.84, -17.87 | 5.51, 43.41, -18.24 |
| FO | Frontal operculum cortex | -39.82, 18.29, 4.62 | 41.14, 18.82, 4.75 |
| FOC | Frontal orbital cortex | -29.69, 23.81, -16.49 | 29.31, 23.43, -16.21 |
| H | Heschl’s gyrus | -45.22, -20.04, 7.31 | 46.04, -17.36, 6.89 |
| INS | Insular cortex | -36.42, 1.01, 0.16 | 37.5, 2.65, -0.17 |
| LFP | Lateral Frontal pole | -20, 52, 32 | 22, 52, 32 |
| LG | Lingual gyrus | -12.57, -65.51, -5.45 | 13.93, -62.73, -4.97 |
| MFP | Medial Frontal pole | -24, 62, 0 | 28, 60, 0 |
| OF | Occipital fusiform gyrus | -26.33, -76.86, -13.45 | 27.24, -75.48, -12.29 |
| OFP | Orbital Frontal pole | -28, 46, -14 | 32, 46, -14 |
| OLi | Lateral occipital cortex | -45.21, -75.63, -1.94 | 45.47, -74.11, -1.51 |
| OLs | Lateral occipital cortex | -32.05, -72.77, 37.99 | 33.03, -71.06, 39 |
| OP | Occipital pole | -17.23, -96.36, 7.23 | 18.09, -95.14, 8.35 |
| PAC | Paracingulate gyrus | -6.82, 36.58, 20.93 | 7.07, 36.37, 22.84 |
| PCN | Precuneus cortex | -8.16, -60.06, 37.25 | 9.33, -58.48, 38.1 |
| PHa | Parahippocampal gyrus | -21.68, -9.28, -30.7 | 22.59, -8.04, -30.63 |
| PHp | Parahippocampal gyrus | -22.15, -32.31, -17.13 | 22.95, -30.25, -16.98 |
| PO | Parietal operculum cortex | -48.4, -31.53, 20.3 | 48.86, -27.69, 21.65 |
| POG | Postcentral gyrus | -38.53, -27.78, 51.5 | 37.25, -26.58, 52.93 |
| PP | Planum polare | -46.77, -5.34, -7.54 | 48.11, -3.56, -7.1 |
| PRG | Precentral gyrus | -34.28, -11.71, 49.18 | 35.08, -10.59, 49.79 |
| PT | Planum temporale | -52.64, -29.69, 10.8 | 54.84, -25.33, 12.39 |
| SC | Subcallosal cortex | -5.7, 20.6, -15.68 | 5.66, 20.42, -15.9 |
| SGa | Supramarginal gyrus | -57, -32.5, 36.94 | 58.23, -27.26, 38.18 |
| SGp | Supramarginal gyrus | -54.86, -46.04, 33.58 | 55.23, -40.29, 33.9 |
| SMC | Juxtapositional lobule cortex | -5.79, -2.67, 56.3 | 6.39, -2.85, 57.64 |
| SPL | Superior parietal lobule | -29.28, -49.4, 57.63 | 29.08, -47.79, 58.92 |
| T1a | Superior temporal gyrus | -56, -3.79, -8.14 | 57.22, -0.98, -10.41 |
| T1p | Superior temporal gyrus | -62.37, -29.14, 3.86 | 61.35, -23.87, 1.5 |
| T2a | Middle temporal gyrus | -57.8, -4.41, -22.05 | 57.86, -1.74, -24.52 |
| T2p | Middle temporal gyrus | -60.95, -27.39, -10.91 | 60.97, -22.35, -12.18 |
| T3a | Inferior temporal gyrus | -47.97, -5.1, -39.12 | 46.31, -2.16, -41.18 |
| T3p | Inferior temporal gyrus | -53.46, -28.21, -26 | 53.8, -23.36, -28.1 |
| TFa | Temporal fusiform cortex | -32.3, -4.53, -41.6 | 30.87, -2.55, -42.28 |
| TFp | Temporal fusiform cortex | -36.02, -29.45, -25.04 | 36.53, -23.81, -28.05 |
| TO2 | Middle temporal gyrus | -57.4, -52.7, 0.87 | 58.32, -49.3, 1.53 |
| TO3 | Inferior temporal gyrus | -51.81, -53.45, -16.68 | 54.19, -49.71, -16.86 |
| TOF | Temporal occipital fusiform cortex | -33.32, -53.65, -15.95 | 35.02, -49.88, -16.56 |
| TP | Temporal pole | -40.44, 11.07, -29.78 | 40.99, 12.93, -29.31 |
| Cau | Caudate nucleus | -10.00, 12.00, 6.00 | 12.00, 14.00, 8.00 |
| Put | Putamen | -28.00, -4.00, 2.00 | 28.00, -4.00, 4.00 |
| Pall | Pallidum | -20.00, -4.00, -2.00 | 20.00, -4.00, -2.00 |
| Thal | Thalamus | -10.00, -20.00, 8.00 | 12.00, -20.00, 8.00 |
| Hipp | Hippocampus | -26.00, -16.00, -20.00 | 28.00, -14.00, -20.00 |
| Amyg | Amygdala | -24.00, -4.00, -18.00 | 24.00, -2.00, -20.00 |

**5 Number of edges at the three edge ranges**

Using the brain parcellation given in supplementary Table S1, the length of all possible edges was catalogued in short, middle and long (short 2.82mm-56.63mm, middle 56.63mm-110.43mm and long 110.43mm-164.23mm according to the MNI152 space). Edges at each length range were counted at all edge densities analysed.


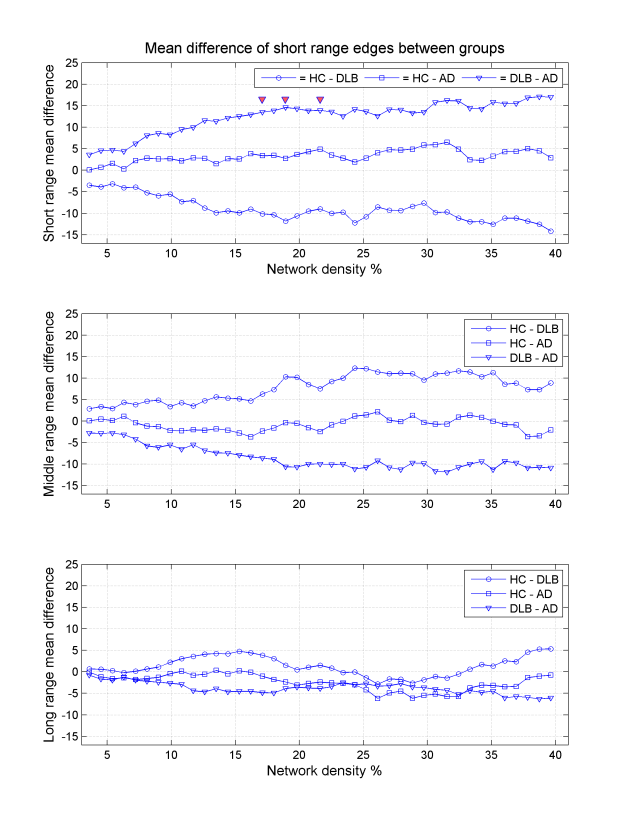
Differences in the number of edges were only significant in the short range between DLB and AD groups at three densities; 17.1%, 18.9%, and 21.6% (supplementary Fig. S1). In average the DLB group showed more short range edges than ADs and healthy controls as shown in supplementary Fig. S1.

Supplementary Figure S1. Lines show the difference in the number of edges between both indicated groups; dementia with Lewy bodies (DLB), Alzheimer’s disease (AD) or healthy controls (HC). For example, the DLB-AD line, shown with the triangular markers, represents the difference between the number of edges in DLB minus the number of edges in AD.

**6 Consistency S_i_ and results for S_i_>10**

The index *S_i_* was designed to study the frequency or consistency of significant differences in the nodal measures. Since *S_i_* was evaluated though all network densities from an average node degree of 4 to 44, the minimum value it can be is 0 (which would mean that node *i* was not significantly different at any of the evaluated densities) and maximum of 41. Hence a value of *S_i_*=41 for a node *i* would mean that this node was significantly different between the two studied groups at all network densities. The way consistency, *S,* is estimated is schematically explained in supplementary Fig. S2.


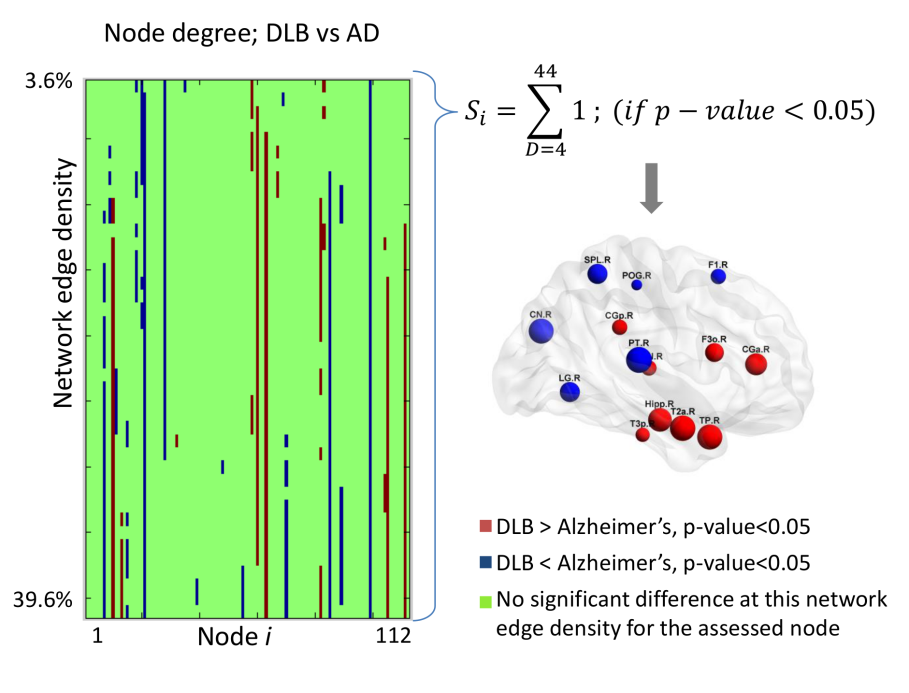


Supplementary Figure S2. *Estimation of consistency S.*

Supplementary Tables S2, S3 and S4 show same results as in Fig. 4 in the main document for nodal differences and for consistency values *S_i_*>10. Colours used in Tables S2, S3 and S4 are coded to be in agreement to the sphere colours (red and blue) in Fig. 4 in main document. For ROI coordinates refer to supplementary Table S1.

**Supplementary Table S2**; Nodal comparisons for consistency ***S_i_***, DLB vs AD. Red colour means DLB>AD while blue means AD>DLB**.**

| **Betweenness Centrality** | | |
| --- | --- | --- |
| ***S*** | ROI |  |
| 37 | CGp.R | Right Posterior cingulate cortex |
| 29 | CGm.L | Left middle posterior cingulate cortex |
| 28 | Hipp.L | Left Hippocampus |
| 25 | F3o.R | Right inferior frontal gyrus |
| 10 | CO.R | Right central opercular cortex |
| 14 | CN.R | Right cuneal cortex |
| 13 | TO2.L | Left middle temporal gyrus |
| 13 | PT.R | Right planum temporale |
| 12 | F1.R | Right superior frontal gyrus |
| **Clustering Coefficient** | | |
| ***S*** | ROI |  |
| 38 | CALC.R | Right intracalcarine cortex |
| 36 | SMC.R | Right juxtapositional lobule cortex |
| 29 | CALC.L | Left intracalcarine cortex |
| 25 | F3t.L | Left inferior frontal gyrus |
| 24 | CGm.R | Right middle cingulate gyrus |
| 23 | INS.L | Left insular cortex |
| 23 | CGm.L | Left middle cingulate gyrus |
| 22 | SMC.L | Left juxtapositional lobule cortex |
| 22 | PRG.R | Right precentral gyrus |
| 20 | CGp.L | Left posterior cingulate cortex |
| 20 | PHp.L | Left Parahippocampal gyrus |
| 20 | PT.L | Left planum temporale |
| 20 | PT.R | Right planum temporale |
| 19 | TO3.L | Left inferior temporal gyrus |
| 19 | CGp.R | Right posterior cingulate gyrus |
| 18 | F2.R | Right middle frontal gyrus |
| 17 | F3o.L | Left inferior frontal gyrus |
| 17 | CN.L | Left cuneal cortex |
| 17 | LG.R | Right lingual gyrus |
| 14 | INS.R | Right insular cortex |
| 14 | FO.R | Right frontal operculum cortex |
| 14 | PO.R | Right parietal operculum cortex |
| 13 | H.L | Left Heschl’s gyrus |
| 13 | Pall.R | Right pallidum |
| 11 | T1a.L | Left superior temporal gyrus |
| 11 | OLi.L | Left lateral occipital cortex |
| 11 | LG.L | Left lingual gyrus |
| **Node degree** | | |
| ***S*** | ROI |  |
| 37 | T2a.R | Right middle temporal gyrus |
| 35 | TP.R | Right temporal pole |
| 31 | TP.L | Left temporal pole |
| 30 | Hipp.L | Left hippocampus |
| 26 | Hipp.R | Right Hippocampus |
| 18 | CGa.R | Right anterior cingulate cortex |
| 41 | PT.R | Right planum temporale |
| 40 | SGa.L | Left supramarginal gyrus |
| 34 | CN.R | Right cuneal cortex |
| 29 | SMC.L | Left juxtapositional lobule cortex |
| 26 | F3t.L | Left inferior frontal gyrus |
| 12 | SPL.R | Right superior parietal lobule |
| 12 | LG.R | Right lingual gyrus |
| 11 | SPL.L | Left superior parietal lobule |

**Supplementary Table S3**; Nodal comparisons for consistency ***S_i_***, Healthy controls vs DLB. Red means Controls>DLB and blue means DLB>Controls.

| **Betweenness Centrality** | | |
| --- | --- | --- |
| ***S*** | ROI |  |
| 30 | T1a.L | Left superior temporal gyrus |
| 21 | PT.L | Left paracingulate gyrus |
| 18 | TO2.L | Left middle temporal gyrus |
| 18 | TO2.R | Right middle temporal gyrus |
| 16 | T2a.R | Right middle temporal gyrus |
| 15 | SPL.L | Left superior parietal lobule |
| 14 | H.R | Right Heschl’s gyrus |
| 40 | F3o.R | Right inferior frontal gyrus |
| 19 | OFP.R | Right orbital frontal pole |
| 19 | F3t.R | Right inferior frontal gyrus |
| **Clustering Coefficient** | | |
| ***S*** | ROI |  |
| 36 | SMC.R | Right juxtapositional lobule cortex |
| 29 | SGp.L | Left supramarginal gyrus |
| 29 | PAC.R | Right paracingulate gyrus |
| 26 | CGm.L | Left middle cingulate gyrus |
| 24 | T3a.L | Left inferior temporal gyrus |
| 19 | FOC.L | Left frontal orbital cortex |
| 19 | PO.R | Right parietal operculum cortex |
| 19 | PT.R | Right planum temporale |
| 18 | PRG.R | Right precentral gyrus |
| 17 | CGp.R | Right posterior cingulate gyrus |
| 15 | PHp.L | Left parahippocampal gyrus |
| 15 | CO.R | Right central opercular cortex |
| 14 | SMC.L | Left juxtapositional lobule cortex |
| 14 | H.L | Left Heschl’s gyrus |
| 12 | INS.L | Left insular cortex |
| 11 | CGa.L | Left anterior cingulate gyrus |
| 11 | T3p.R | Right inferior temporal gyrus |
| 13 | FMC.R | Right frontal medial cortex |
| **Node degree** | | |
| ***S*** | ROI |  |
| 41 | T1a.L | Left superior temporal gyrus |
| 41 | SPL.L | Left superior parietal lobule |
| 41 | PT.L | Left planum temporale |
| 41 | PT.R | Right planum temporale |
| 26 | H.L | Left Heschl’s gyrus |
| 26 | TO2.R | Right middle temporal gyrus |
| 21 | LG.R | Right lingual gyrus |
| 19 | SPL.R | Right superior parietal lobule |
| 18 | T1p.R | Right superior temporal gyrus |
| 15 | TO2.L | Left middle temporal gyrus |
| 12 | CN.R | Right cuneal cortex |
| 41 | F3t.R | Right inferior frontal gyrus |
| 41 | F3o.R | Right inferior frontal gyrus |
| 36 | OLs.R | Right lateral occipital cortex |
| 35 | LFP.R | Right lateral frontal pole |
| 29 | Hipp.L | Left hippocampus |
| 22 | FMC.R | Right frontal medial cortex |
| 21 | Thal.R | Right thalamus |
| 17 | PHa.R | Right parahippocampal gyrus |
| 16 | TP.R | Right temporal gyrus |
| 12 | SC.R | Right subcallosal cortex |
| 11 | MFP.L | Left medial frontal pole |

**Supplementary Table S4**; Nodal comparisons for consistency ***S_i_***, Controls vs AD. Red means Controls>AD and blue means AD>Controls

| **Betweenness Centrality** | | | |
| --- | --- | --- | --- |
| ***S*** | ROI |  |  |
| 31 | CGm.L |  | Left middle cingulate gyrus |
| 20 | T2a.R |  | Right middle temporal gyrus |
| 15 | T1a.R |  | Right superior temporal gyrus |
| 13 | PT.L |  | Left planum temporale |
| 11 | CGp.R |  | Right posterior cingulate gyrus |
| 17 | F3o.R |  | Right inferior frontal gyrus |
| 10 | PAC.R |  | Right paracingulate gyrus |
| **Clustering Coefficient** | | | |
| ***S*** | ROI |  |  |
| 33 | T3a.L |  | Left inferior temporal gyrus |
| 12 | PAC.R |  | Right paracingulate gyrus |
| 11 | FOC.L |  | Left frontal orbital cortex |
| 11 | FOC.R |  | Right frontal orbital cortex |
| **Node degree** | | | |
| ***S*** | ROI |  |  |
| 37 | T2a.R |  | Right middle temporal gyrus |
| 15 | INS.L |  | Left insular cortex |
| 13 | CGm.L |  | Left middle cingulate gyrus |
| 41 | F3t.L |  | Left inferior frontal gyrus |
| 41 | OLs.R |  | Right lateral occipital cortex |
| 39 | SMC.L |  | Left juxtapositional lobule cortex |

**7 Correlation trends between cognitive scores and network measures in AD**

Contrary to our DLB group where we found significant correlations between cognitive and fluctuating attention clinical scores and integrated global network measures, in AD we were unable to find significant correlations. We attributed this to two factors in our AD group: First, our AD patients had relatively mild cognitive impairment which can be seen by their high scores in the MMSE compared to previously published studies ([Liu, et al., 2013](#_ENREF_11),[Stam, et al., 2009](#_ENREF_17)). In addition to this, our AD patients were taking cholinesterase inhibitor (AChEI) treatment which previous research has proved to restore functional connectivity in mild AD patients ([Li, et al., 2012](#_ENREF_9)).

**
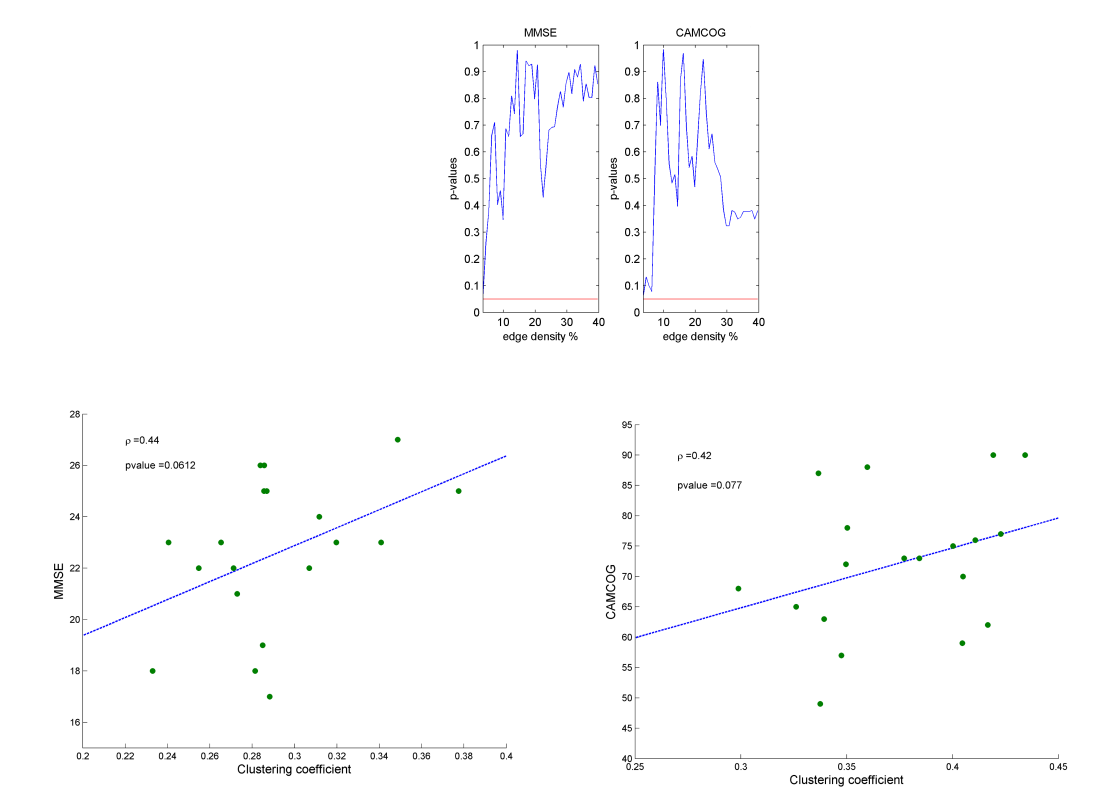
**

Supplementary Figure S3. Correlation trends between clustering coefficient and MMSE/CAMCOG in AD. The MMSE showed a trend at 3.6% edge density (p-value=0.061) while the CAMCOG showed a trend at 6.3% density (p-value=0.077). The top figures show p-values at each of the assessed densities. The lowest p-values are found at low edge densities.


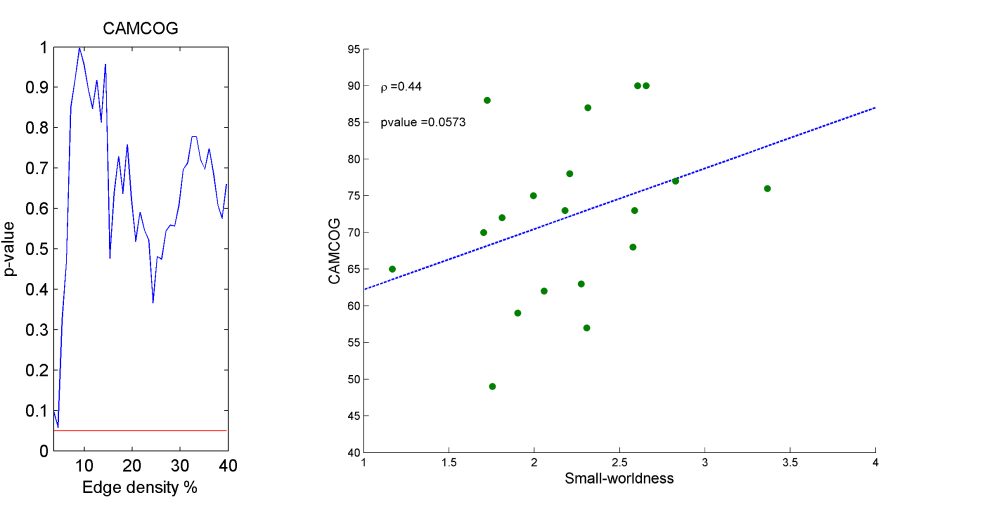


Supplementary Figure S4. Correlation trends between CAMCOG and small-worldness in AD. This trend was found at edge density 4.5% (p-value=0.057).

From our AD group, we were only able to find correlation trends between network measures and cognitive clinical scores (supplementary Fig. S3 and S4). Specifically, CAMCOG showed a trend in correlation with clustering coefficient at edge density 6.3% (p-value=0.077) and with small-worldness at density 4.5% (p-value=0.057). The MMSE and clustering coefficient also showed a trend in correlation at density 3.6% (p-value=0.061). These correlation trends were found at very low densities, which mean that dysfunctions in the strongest connections in the functional network were correlated with cognitive deficits in AD. This agrees with previous reports where correlations are found between cognitive clinical scores such as the MMSE and network measures at low densities ([Ciftci, 2011](#_ENREF_3),[Liu, et al., 2013](#_ENREF_11)). For instance, Liu et al ([Liu, et al., 2013](#_ENREF_11)) reported significant correlations between the MMSE and clustering coefficient and efficiency at the edge density of 1%. In addition, Ciftci et al. ([Ciftci, 2011](#_ENREF_3)) found alterations in AD by analysing the functional minimum spanning tree network, which is a network where all nodes are connected with the minimum number of edges, a feature that occurs at very low edge densities. Furthermore, findings at very low edge densities are commonly reported in AD literature ([Chen, et al., 2013](#_ENREF_2),[Sanz-Arigita, et al., 2010](#_ENREF_15),[Zhao, et al., 2012](#_ENREF_23)). Hence, our results in AD for correlations with clinical scores, albeit not being significant at the range of edge densities chosen in our study, are in line with previously reported findings in AD research.

**8 Higher small-worldness in DLB compared to healthy controls and AD**

One the findings in our study was the higher small-worldness σ in DLB which results at first glance contradictory to what one might expect in a neurodegenerative disease, as typically this network measure is associated in the literature with higher efficiency and with a healthier brain network.

However, higher small-worldness is not necessarily equivalent to a healthier brain network. This measure which is widely used in AD research is severely affected by the node distribution of the real network $G$ and by the clustering coefficient of the random equivalent network $G_{rand}$ needed to estimate σ, as previously reported in ([van Wijk, et al., 2010](#_ENREF_22)) and discussed in much more detail in ([Telesford, et al., 2011](#_ENREF_21)). The sensitivity of small-worldness to these variables thus might cause two topologically different networks with same edge density have equal small-worldness ([Telesford, et al., 2011](#_ENREF_21)).

To clarify discrepancies in small-worldness, Telesford et al. ([Telesford, et al., 2011](#_ENREF_21)) proposed a new measure named Omega (ω) which is mathematically defined as:

$$\omega=\frac{L_{rand}}{L}-\frac{C}{C_{latt}}$$

Where $C_{latt}$ is the clustering coefficient of the equivalent lattice network of the real network ([Sporns and Zwi, 2004](#_ENREF_16)) in a similar way as $C_{rand}$ is the clustering coefficient of the equivalent random network in the small-world index σ. The index ω categorizes a network as small-world if the index is close to zero, with an additional advantage: if ω is negative, the network is biased to a lattice topology while a positive value will mean that the network is biased towards a random topology. Furthermore, ω is bounded between -1 and 1 which makes it easier to compare between networks even if these are of different size ([Telesford, et al., 2011](#_ENREF_21)).

Omega results for our three study groups are shown in supplementary Fig. S5 and to estimate $C_{latt}$ we latticized each real network 50 times and estimate their average clustering $C_{latt}$. For this we used the latticization function from the Brain Connectivity Toolbox ([Rubinov and Sporns, 2010](#_ENREF_13)) which preserves the node degree distribution, with 5 rewirings per edge. Supplementary Fig. S5 shows that for edge densities below 9% the three groups show a more lattice topology while at higher densities all groups show a more random topology. Telesford et al. ([Telesford, et al., 2011](#_ENREF_21)) suggested that a small-world network should be -0.1<ω<0.1 which is between 7.2% and 12% for healthy controls and agrees with the current literature in brain connectivity. Interestingly for lower densities (<9%) our DLB group showed, on average, a topology closer to lattices than ADs and healthy controls while at higher densities, >9%, the DLB group shows a more random topology than ADs and healthy controls (blue line in Fig. S5). This result agrees with our observations of a regularization of the DLB functional network caused by the fact that short range connections appear to be relatively stronger in DLB (Fig. 2 in main document) and thus these short range edges manifest as a lattice at low edge densities.


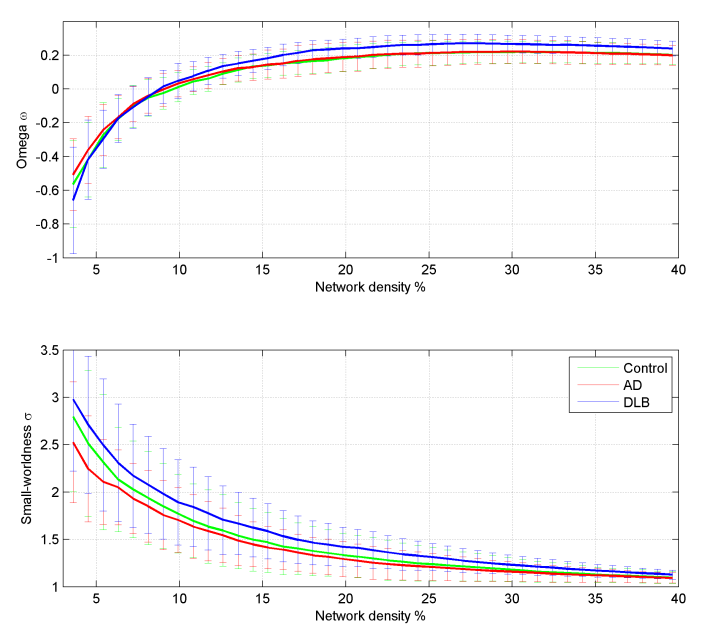


Supplementary Figure S5. Omega ω group averages for the three study groups and small-worldness values are reproduced for comparisons. Alzheimer’s disease, AD; Dementia with Lewy bodies, DLB.

The observation that at higher edge densities the functional networks of our three groups (healthy controls, DLBs and ADs) show a more random topology is certainly expected since edges with low correlation values are being assumed as true edges, whereas some of these might be a result of natural noise in the fMRI recordings leading to a more random topology. However, the DLB group showed a slightly higher randomness than healthy controls and AD patients. This higher randomness might be associated to the disease and the lower neural synchronizations in DLB, possibly as a result of the known pre-synaptic dysfunctions caused by α-synuclein aggregates which alter in more degree middle and long distance edges (Fig. 2 in main document).

Motion in our patients is a confound that might cause these results, but our three groups did not show significant differences in their motion parameters as explained in Section 2 in this supplementary document about motion parameters. Furthermore our exclusion criteria for motion were strict (<2mm for translation and <1° for rotation) and motion related independent components were filtered from the datasets using independent component analysis (see Methods section in main document). In addition to this our DLB group moved, on average, less than the AD group as shown in Section 2 in this supplementary document. Hence, motion cannot explain the differences found between AD and DLB groups in our study.

To clarify the higher small-worldness observed in the DLB group, we performed an analysis similar to Telesford et al ([Telesford, et al., 2011](#_ENREF_21)). We estimated correlations between small-worldness $\sigma$ and the network measures needed to estimate the $\sigma$ index; the raw clustering coefficient $C$ of the real network, the raw characteristic path length $L$, same two measures from the random equivalent network ($L_{rand}$, $C_{rand}$) and we also included the normalized versions whose ratio is used to estimate small-worldness ($L_{norm}$, $C_{norm}$). For this analysis we used integrated measures by estimating the average through all edge densities (from 3.6% to 39.7%).

As expected small-worldness was highly influenced by the clustering of the random equivalent network $C_{rand}$, as shown in supplementary Fig. S6 (first and third column).

Notably as shown at the bottom row of Supplementary Fig. S6, the small-world index of the healthy control group showed significant correlations with all network measures except the characteristic path length of the random equivalent network $L_{rand}$. On the contrary, this did not happen in both patient groups (DLB or AD); patients demonstrated significant correlation with the random clustering coefficient $C_{rand}$ but not with the original raw network measures *C* and *L*. This suggests that in the healthy control group, the measures of the real network *C* and *L* have a degree of influence in the small-world index of a healthy brain, while in a diseased brain, small-world indices should be taken cautiously since these small-world values depend mostly on the clustering of the random equivalent network. Certainly, this observation will require further research and corroboration in independent cohorts.

Finally, our analysis on small-worldness indicates that the higher small-woldness observed in DLB results as consequence of the low clustering coefficient values of the random equivalent network $C_{rand}$ which has equal node degree distribution of the real networks in the DLB group.


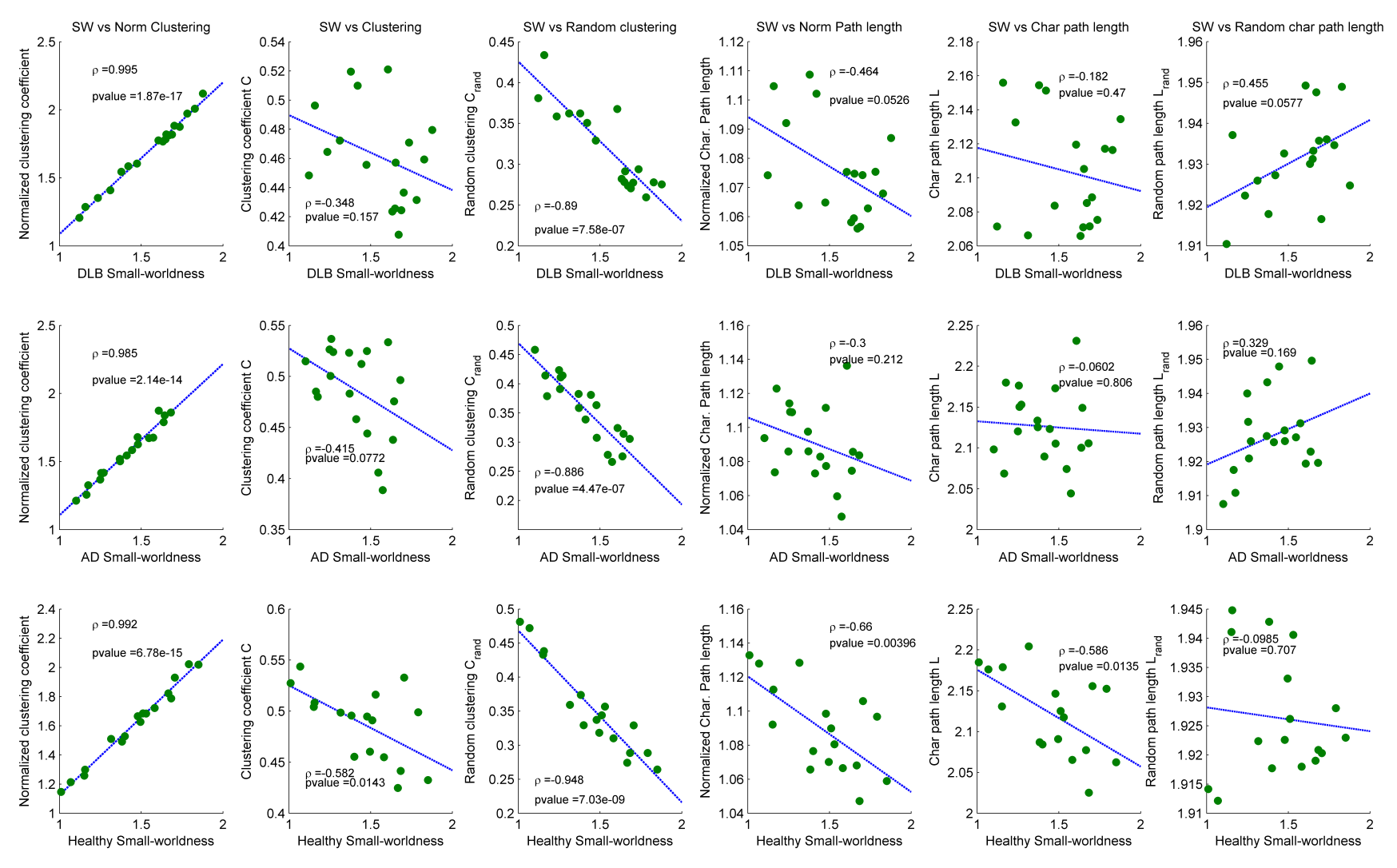


Supplementary Figure S6. Influence of the network measures in the small-worldness index for the three groups. From top to bottom row; Dementia with Lewy bodies (DLB), Alzheimer’s disease (AD), and healthy controls. The figure shows correlations between integrated small-world index for the three groups and network measures needed to estimate small-worldness; clustering coefficient, characteristic path length, the random network equivalents of these measures and the normalized measures.

**9 Local correlations of Parkinsonism and visual hallucination clinical scores**

We were not able to find significant correlations between the integrated global network measures and the clinical scores for Parkinsonism (UPDRS) and the frequency/severity of visual hallucinations (NPI^hall^) in DLB. However, as explained in the main document a possibility for this is that the patho-aetiology of these core symptoms is more regionally specific; e.g. visual area and motor system dysfunctions for hallucinations ([Taylor, et al., 2011](#_ENREF_19),[Taylor, et al., 2012](#_ENREF_20)) and Parkinsonism ([Stoessl, et al., 2014](#_ENREF_18)) respectively.

To test this idea we integrated the local network measures of node degree, nodal clustering coefficient and nodal betweenness centrality by averaging across all edge densities analysed in our study (from 3.6% to 39.7%, see section 3 in this supplementary material). As a result, we obtained one integrated nodal measure per node and per participant which we subsequently correlated using Spearman’s correlation (Matlab) with the UPDRS and NPI^hall^ clinical scores.

Results for this exploratory analysis are shown in Supplementary Table S5 at p-value<0.01 for significance.

**Supplementary Table S5.** Integrated nodal network measures that resulted significant with clinical scores for Parkinsonism (UPDRS) and visual hallucinations (NPI^hall^) at p-value<0.01.

| **NPI^hall^ and node degree** | P-value | R |
| --- | --- | --- |
| L Postcentral gyrus | 0.0083 | -0.600 |
| L Putamen | 0.0080 | 0.602 |
| **NPI^hall^ – nodal betweenness centrality** |  |  |
| R Intracalcarine cortex | 0.0037 | -0.645 |
| R Temporal occipital fusiform cortex | 0.0054 | 0.626 |
| **UPDRS – node degree** |  |  |
| L Angular gyrus | 0.0040 | 0.642 |
| L Lateral occipital cortex | 0.0074 | 0.608 |
| R Medial Frontal pole | 0.0017 | -0.684 |
| **UPDRS – nodal clustering coefficient** |  |  |
| L Caudate | 0.0039 | 0.644 |
| **UPDRS – nodal betweenness centrality** |  |  |
| R Orbital Frontal pole | 0.0064 | -0.616 |

The NPI^hall^ score showed positive correlations with the node degree of the left postcentral gyrus and the nodal betweenness centrality of the right temporal occipital fusiform cortex. Also, negative correlations with the node degree of the left postcentral gyrus and the nodal betweenness centrality of the right intracalcarine cortex were found for this clinical score.

Although these analyses are purely exploratory, it is interesting to note that the severity/frequency of visual hallucinations was negatively correlated with the betweenness centrality of the intracalcarine cortex. Betweenness centrality is a measure of node reachability which suggests that visual hallucination severity and frequency are correlated with an intracalcarine cortex that is less connected from the rest of the brain functional network. The contrary logic can be applied on the right temporal occipital fusiform cortex whose betweenness centrality is positively correlated with NPI^hall^, i.e. visual hallucinations are correlated with a more connected or accessible temporal occipital fusiform cortex; this resonates with visual hallucination models which indicate the primacy of an unregulated ventral visual stream in inappropriately producing visual prototypes which manifest as visual hallucinations ([Collerton, et al., 2005](#_ENREF_4),[Sanchez-Castaneda, et al., 2010](#_ENREF_14)).

Furthermore the left caudate nucleus resulted positively correlated with the UPDRS for clustering coefficient and this basal ganglia node which is part of the striata fits well with the context of known dopamine deficits in these areas with Parkinsonism ([Colloby and O'Brien, 2004](#_ENREF_5),[Stoessl, et al., 2014](#_ENREF_18)).

Furthermore the negative correlations found in frontal lobe regions for node degree and betweenness centrality may reflect intrinsic disturbances in fronto-striatal connectivity which typically occur in Lewy body diseases ([Delli Pizzi, et al., 2014](#_ENREF_7),[Sanchez-Castaneda, et al., 2010](#_ENREF_14)). Overall these analyses and their findings will require further research and validation with different fMRI techniques/methods that highlight the relation of functional alterations in the brain with the core symptoms of DLB.

References

Bohr, I.J., Kenny, E., Blamire, A., O'Brien, J.T., Thomas, A.J., Richardson, J., Kaiser, M. 2012. Resting-state functional connectivity in late-life depression: higher global connectivity and more long distance connections. Frontiers in psychiatry 3, 116. doi:10.3389/fpsyt.2012.00116.

Chen, G., Zhang, H.-Y., Xie, C., Chen, G., Zhang, Z.-J., Teng, G.-J., Li, S.-J. 2013. Modular reorganization of brain resting state networks and its independent validation in Alzheimer's disease patients. Frontiers in human neuroscience 7, 456. doi:10.3389/fnhum.2013.00456.

Ciftci, K. 2011. Minimum spanning tree reflects the alterations of the default mode network during Alzheimer's disease. Annals of biomedical engineering 39(5), 1493-504. doi:10.1007/s10439-011-0258-9.

Collerton, D., Perry, E., McKeith, I. 2005. Why people see things that are not there: A novel Perception and Attention Deficit model for recurrent complex visual hallucinations. Behavioral and Brain Sciences 28(06), 737-57. doi:doi:10.1017/S0140525X05000130.

Colloby, S., O'Brien, J. 2004. Functional imaging in Parkinson's disease and dementia with Lewy bodies. Journal of geriatric psychiatry and neurology 17(3), 158-63. doi:10.1177/0891988704267468.

De Vico Fallani, F., Richiardi, J., Chavez, M., Achard, S. 2014. Graph analysis of functional brain networks: practical issues in translational neuroscience. Philosophical Transactions of the Royal Society of London B: Biological Sciences 369(1653).

Delli Pizzi, S., Maruotti, V., Taylor, J.P., Franciotti, R., Caulo, M., Tartaro, A., Thomas, A., Onofrj, M., Bonanni, L. 2014. Relevance of subcortical visual pathways disruption to visual symptoms in dementia with Lewy bodies. Cortex 59C, 12-21. doi:10.1016/j.cortex.2014.07.003.

Gießing, C., Thiel, C.M., Alexander-Bloch, A.F., Patel, A.X., Bullmore, E.T. 2013. Human Brain Functional Network Changes Associated with Enhanced and Impaired Attentional Task Performance. The Journal of Neuroscience 33(14), 5903-14. doi:10.1523/jneurosci.4854-12.2013.

Li, W., Antuono, P.G., Xie, C., Chen, G., Jones, J.L., Ward, B.D., Franczak, M.B., Goveas, J.S., Li, S.-J. 2012. Changes in regional cerebral blood flow and functional connectivity in the cholinergic pathway associated with cognitive performance in subjects with mild Alzheimer's disease after 12-week donepezil treatment. NeuroImage 60(2), 1083-91. doi:<http://dx.doi.org/10.1016/j.neuroimage.2011.12.077>.

Liao, W., Chen, H., Feng, Y., Mantini, D., Gentili, C., Pan, Z., Ding, J., Duan, X., Qiu, C., Lui, S., Gong, Q., Zhang, W. 2010. Selective aberrant functional connectivity of resting state networks in social anxiety disorder. NeuroImage 52(4), 1549-58. doi:<http://dx.doi.org/10.1016/j.neuroimage.2010.05.010>.

Liu, Y., Yu, C., Zhang, X., Liu, J., Duan, Y., Alexander-Bloch, A.F., Liu, B., Jiang, T., Bullmore, E. 2013. Impaired Long Distance Functional Connectivity and Weighted Network Architecture in Alzheimer's Disease. Cerebral cortex. doi:10.1093/cercor/bhs410.

Power, J.D., Fair, D.A., Schlaggar, B.L., Petersen, S.E. 2010. The development of human functional brain networks. Neuron 67(5), 735-48. doi:10.1016/j.neuron.2010.08.017.

Rubinov, M., Sporns, O. 2010. Complex network measures of brain connectivity: uses and interpretations. NeuroImage 52(3), 1059-69. doi:10.1016/j.neuroimage.2009.10.003.

Sanchez-Castaneda, C., Rene, R., Ramirez-Ruiz, B., Campdelacreu, J., Gascon, J., Falcon, C., Calopa, M., Jauma, S., Juncadella, M., Junque, C. 2010. Frontal and associative visual areas related to visual hallucinations in dementia with Lewy bodies and Parkinson's disease with dementia. Movement Disorders 25(5), 615-22. doi:10.1002/mds.22873.

Sanz-Arigita, E.J., Schoonheim, M.M., Damoiseaux, J.S., Rombouts, S.A., Maris, E., Barkhof, F., Scheltens, P., Stam, C.J. 2010. Loss of 'small-world' networks in Alzheimer's disease: graph analysis of FMRI resting-state functional connectivity. PloS one 5(11), e13788. doi:10.1371/journal.pone.0013788.

Sporns, O., Zwi, J.D. 2004. The small world of the cerebral cortex. Neuroinformatics 2(2), 145-62. doi:10.1385/ni:2:2:145.

Stam, C.J., de Haan, W., Daffertshofer, A., Jones, B.F., Manshanden, I., van Cappellen van Walsum, A.M., Montez, T., Verbunt, J.P.A., de Munck, J.C., van Dijk, B.W., Berendse, H.W., Scheltens, P. 2009. Graph theoretical analysis of magnetoencephalographic functional connectivity in Alzheimer's disease. Brain : a journal of neurology 132(1), 213-24. doi:10.1093/brain/awn262.

Stoessl, A.J., Lehericy, S., Strafella, A.P. 2014. Imaging insights into basal ganglia function, Parkinson's disease, and dystonia. The Lancet 384(9942), 532-44. doi:10.1016/s0140-6736(14)60041-6.

Taylor, J.P., Firbank, M., Barnett, N., Pearce, S., Livingstone, A., Mosimann, U., Eyre, J., McKeith, I.G., O'Brien, J.T. 2011. Visual hallucinations in dementia with Lewy bodies: transcranial magnetic stimulation study. The British journal of psychiatry : the journal of mental science 199(6), 492-500. doi:10.1192/bjp.bp.110.090373.

Taylor, J.P., Firbank, M.J., He, J., Barnett, N., Pearce, S., Livingstone, A., Vuong, Q., McKeith, I.G., O'Brien, J.T. 2012. Visual cortex in dementia with Lewy bodies: magnetic resonance imaging study. The British journal of psychiatry : the journal of mental science 200(6), 491-8. doi:10.1192/bjp.bp.111.099432.

Telesford, Q.K., Joyce, K.E., Hayasaka, S., Burdette, J.H., Laurienti, P.J. 2011. The ubiquity of small-world networks. Brain connectivity 1(5), 367-75.

van Wijk, B.C.M., Stam, C.J., Daffertshofer, A. 2010. Comparing Brain Networks of Different Size and Connectivity Density Using Graph Theory. PloS one 5(10), e13701. doi:10.1371/journal.pone.0013701.

Zhao, X., Liu, Y., Wang, X., Liu, B., Xi, Q., Guo, Q., Jiang, H., Jiang, T., Wang, P. 2012. Disrupted Small-World Brain Networks in Moderate Alzheimer's Disease: A Resting-State fMRI Study. PloS one 7(3), e33540. doi:10.1371/journal.pone.0033540.
